# The effect of family boundary flexibility on employees’ work engagement: a study based on person-environment fit theory perspective

**DOI:** 10.3389/fpsyg.2023.1185239

**Published:** 2023-09-28

**Authors:** Dexiang Yang, Yakun Liu, Huiqin Zhang, Yuxiang Zhang

**Affiliations:** College of Management Science, Chengdu University of Technology, Chengdu, China

**Keywords:** family boundary flexibility, family-work enrichment, family support, work engagement, person-environment fit, employee resilience, enterprise

## Abstract

Under the impact of the era of big data and public emergency, the blurring of family-work boundaries and the increasing burden of family responsibilities will pose a great challenge to employee resilience and family work balance, which in turn will affect employees’ work engagement. Therefore, based on the person-environment fit theory, this study aims to explore the potential mechanism and boundary conditions of employee family boundary flexibility fit on work engagement. This study conducted a random sampling of enterprise employees in China. A sample of 433 participants completed a questionnaire to provide data. We conduct hierarchical regression and Bootstrap analysis to verify the hypothesis model. The study found that employees’ work engagement is significantly improved when their family boundary flexibility is matched. Family-work enrichment plays a role in mediating the impact of employees’ family boundary flexibility on work engagement. The relationship between family-work enrichment and work engagement is moderated by family support. Therefore, enterprises should respect and value each employee’s family boundary flexibility, establish family-friendly policies, and consider personal family boundary flexibility in employees’ career development planning. This will promote the enhancement of employee resilience, enable better engagement in work, improve work efficiency, and enhance the core competitiveness of enterprises.

## 1. Introduction

With the advancement of the digital economy and the widespread adoption of artificial intelligence, enterprises also have become more flexible in their employment patterns (Wang et al., 2022). COVID-19 makes remote working become the norm overnight (Kniffin et al., 2021). It results in an increasingly blurred and flexible boundary between employees’ work and home domains (Allen et al., 2021; Kerman et al., 2022). This has undoubtedly posed a serious challenge to employees in terms of employee resilience and balancing and managing home and work. In the face of expanding family responsibility and the multiple family roles, organizations are beginning to worry that employees will reduce their work engagement because they spend more energy on their family affairs. It is found that resilience can help employees cope with challenges, overcome difficulties, quickly restore balance, and achieve growth and development (King et al., 2016; Linnenluecke, 2017; Xie et al., 2023).

The Aon’s report “THE RISING RESILIENT: How workforce resilience will enable businesses to thrive,” released in 2022, identifies workforce resilience as a key competency for organizations to cope with crises and improve their overall business outcomes. The report builds a workforce resilience model by analyzing 10 factors that influence workforce resilience, with health being a key factor in achieving resilience. Angela Zhou, Vice President of Human Resources at Philips Greater China, said it is important to fully integrate employee health into corporate strategy to build resilient teams. Philips provided employee Assistance Program (EAP) for employees and their families during COVID-19, effectively alleviating employee mental health issues; while implementing family-friendly policies to enable employees to balance work and family. It can be seen that employee resilience is highly positively correlated with work-family balance (Kim and Windsor, 2015). Therefore, how to cross family boundaries and resile from the clutter of family affairs in order to better cope with the challenges at work, and improve the work commitment and happiness of employees, has become an urgent problem in society to solve.

As an important indicator to measure employees’ work enthusiasm and participation, work engagement has always been a hot topic for researchers and managers (Malinowska and Tokarz, 2020). Especially, how to make employees full of passion and vitality and maintain a high level of work engagement. Work engagement is a cognitive state and positive emotion that people have toward the work domain (Schaufeli et al., 2002). Work engagement positively affects employees’ job performance (Shantz et al., 2016; Beltrán-Martín et al., 2022), wellbeing (Junca-Silva et al., 2017; Radic et al., 2020), proactive behavior and voice behavior (Schmitt et al., 2016). Therefore, work engagement significantly contributes to enhancing the organization’s core competitiveness (Chen and Peng, 2021). At present most of these studies have focused on workplace resources and leadership (Cai et al., 2018; Björk et al., 2021), and relatively few have delved into the factors that contribute to job engagement from a family perspective.

Recent research suggests that employee’s family motivation serves as a significant catalyst for exerting diligent efforts in the workplace (Menges et al., 2017; Zhang et al., 2020). Employees will actively transfer home resources to the work field, thereby improving the performance of the work field (Dumas and Stanko, 2017). Based on boundary theory, individuals create distinct boundaries between their work and family domains (Liu et al., 2013). Most current research has focused on work boundary flexibility (Ferguson et al., 2015; Lott, 2020; Urbanavičiūtė et al., 2022; Seeber and Erhardt, 2023), and family boundary flexibility is also a significant part of the family-work interaction and can facilitate employees’ productivity (Babalola et al., 2021; Ren et al., 2022), stimulate their work vitality, which is the key to effectively improve the work involvement of employees (Ammons, 2013). Therefore, the primary focus will be on examining the direct effect of family boundary flexibility on work engagement in this study.

At present, the relationship between work and family is the most important social environment that affects the quality of employees’ work, and there is a mutual spillover effect between them (Lin et al., 2021; Wu et al., 2021). In traditional work-family analyses, a negative perspective based on conflict has been dominant, ignoring the favorable influence between home and work (Spieler et al., 2017; Elahi et al., 2022), but the favorable influence between home and work can facilitate employees’ better engagement at their jobs (Greenhaus and Powell, 2006; Masuda et al., 2012). In consequence, the mediating role of family-work enrichment in the influence of family boundary elasticity on work engagement will be the second issue to be discussed in this study.

Beyond that, Person-environment fit theory suggests that the interaction between individuals and the environmental variable in which he or she lives has a much greater effect on individual behavior than the direct effect of individual and environmental variables on individual behavior (van Vianen, 2018). Family system theory proposes that the actions and attitudes of individuals can be significantly influenced by the attitudes and actions of their family members (Hammer et al., 2005). Therefore, the supportive resources provided to employees by their family domains (Li and Zhao, 2009) allow employees to put aside family concerns and devote themselves to their work, which further increases their resilience to adapt to the challenges and pressures at work (Masten, 2018). In this study, the third issue to be examined will be the moderating role of family support.

## 2. Theories and hypothesis

### 2.1. Family boundary flexibility and work engagement

According to the Person-Environment Fit theory, individual adaptation and wellbeing in a given environment depends on the degree of match between the characteristics of the environment and the characteristics of the individual, thus impacting on individual attitude and behavior (van Vianen, 2018). As an individual characteristic, family boundary flexibility describes the extent to which individuals are cognitively, psychologically and behaviorally separated in the family domain (Kreiner, 2006; Wepfer et al., 2018), and it encompasses family flexibility-willingness and family flexibility-ability. Researchers refer to the people’s self-cognitive evaluation of their ability to leave the family field and satisfy the external environmental characteristics required by the work domain as boundary flexibility ability, and the preference to across from home to work area as family flexibility willingness (Matthews and Barnes-Farrell, 2010; Matthews et al., 2010). If family flexibility-ability and family flexibility-willingness are matched, people will feel positive sensations, thereby further enhancing their positive work experiences (Ma et al., 2014).

Work engagement represents a continuous positive state of individuals in their work setting, which reflects the active involvement and commitment of employees, including three dimensions vitality, dedication, and focus (Schaufeli et al., 2002). Research shows that employees who are highly engaged in their work tend to achieve superior performance outcomes (Jiang et al., 2021). Employees can improve job engagement by changing cognition, relationships and tasks. The higher the matching degree of family flexibility-ability and family flexibility-willingness, the easier it is for employees to cross from the family field to the work field. The increased resilience of employees can help them cope with the stress of their home environment (Liu et al., 2023). So as to stimulate their own work vitality to the maximum extent, and maintain the state of constant growth and learning (Ammons, 2013). In summary, this paper makes the following hypothesis:

Hypothesis 1: Employees’ family boundary flexibility has a positive impact on their work engagement.

### 2.2. The mediating role of family-work enrichment

An individual’s resources from family help them perform better at work, leading to positive attitudes and behavioral reactions, which is known as family-work enrichment (Carlson et al., 2006; Greenhaus and Powell, 2006). Individuals who possess a high degree of matching family boundary flexibility are more inclined to facilitate the flow of supportive resources and their own energy and emotions from the family area to the work area (Yang et al., 2023). It makes people feel that they have more energy to put into their task (Çetin et al., 2022), and increase their own resilience in the work domain, which fosters a greater level of engagement (Ma et al., 2014). On the contrary, it is difficult for individuals with low family boundary flexibility matching to achieve boundary crossing, which limits their role transition from the family area to the work area, hinders the occurrence of family-work enrichment, and thus inhibits employees’ work engagement (Vaziri et al., 2020).

This paper argues that home-work enrichment is a link between the home sphere and the work sphere. Family boundary flexibility allows employees to feel family-work gain through the transfer of resources across borders, so that they can devote themselves to work. In summary, this paper makes the following hypothesis:

Hypothesis 2: Family-work Enrichment acts as a mediating factor between employees’ family boundary flexibility and their work engagement.

### 2.3. The moderating effect of family support

The theory of person-environment fit states, that the behavior of individuals is a result of the interplay between personal traits and external circumstances (Livingstone, 1997; van Vianen, 2018). When the supply of the environment is matched with the needs of individuals, positive experiences such as individual satisfaction and happiness will be enhanced, thus enabling individuals to have more energy and positive emotions to fulfill other role requirements (Chen et al., 2009). Family support is a supportive resource provided by the family environment, including emotional understanding, practical help and information support (Aryee et al., 2005; Michel et al., 2011), which can enhance employees’ vitality at work. The supportive resources from the family are mainly instrumental resources and emotional resources (Aryee et al., 2005; He et al., 2023). For example, family members can help employees take the initiative to share household chores and take care of the elderly and children, which can objectively reduce the time pressure on employees in dealing with family matters, thus ensuring that they can have sufficient energy to devote to work and more time to develop their personal skills and knowledge and increase their work engagement. In addition, positive and effective communication between family members and employees, as emotional support, comfort, and motivation, will make employees feel loved and respected, thus reducing resistance to sad and dysphoric emotions (Ten Brummelhuis and Bakker, 2012; Chen and Ellis, 2021). Family support can enhance employee mental health, reduce stress, and complete challenging tasks (Luthans et al., 2010; Yang et al., 2022), which is conducive to improving employee resilience. In summary, this paper proposes the following hypothesis:

Hypothesis 3: Employees’ family support positively moderates the relation between family-work enrichment and work engagement.

Combining the results of the derivation of hypotheses 1–3 and the mediated model proposed by Edwards and Lambert (2007), we can conclude that employees’ family support plays a moderating role in the mediating impact of family-work enrichment on family boundary flexibility and work engagement. Specifically, while employees receive relatively high levels of family support, the abundance of family resources enables employees to fulfill their family roles better, which frees them up to focus on their careers. In this case, the positive influence of family-work enrichment on work engagement is stronger, leading to higher levels of work engagement for employees. Otherwise, the effect is weaker.

Hypothesis 4: The level of family support positively moderates the mediating role of family-work enrichment between family boundary flexibility and work engagement.

**FIGURE 1:**
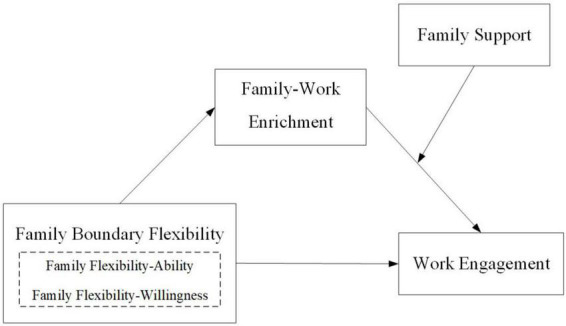
The hypothesis model of the relationships between family boundary flexibility, family-work enrichment, family support, and work engagement.

In light of the previous analysis, we have constructed a theoretical model that includes family boundary flexibility as the independent variable, work engagement as the dependent variable, family-work enrichment as the mediating variable, and family support as the moderating variable. Specifically, family support serves as a moderator, not only influencing the relationship between family-work enrichment and work engagement but also moderating the mediating role of family-work enrichment. The overall research model is demonstrated in Figure 1.

## 3. Method

### 3.1. Participants and procedures

This study is quantitative. The data samples for this study were obtained from reputable third-party professional survey platforms in China, including Credamo,^1^ Wenjuanxing,^2^ and Wenjuanwang.^3^ Credamo has a strong reputation in the academic community, with users who have had their papers accepted in top international journals such as the Journal of Consumer Research and Journal of Business Research (Huang and Sengupta, 2020; Li et al., 2022). According to the data on the official website, Wenjuanxing covers over 3 million enterprises and 90% of universities in China, while Wenjuanwang boasts more than 10,000 partners. As reliable online questionnaire survey platforms, they are widely recognized and trusted by Chinese scholars for collecting survey data. By utilizing these professional research platforms, this study conducted a random sampling of enterprise employees in China and administered electronic questionnaires. These platforms enabled researchers to recruit samples from online platforms and through member recommendations, providing incentives and rewards to encourage participation. To ensure data quality, we excluded questionnaires that were extremely short in response time, contained missing information, or displayed obvious regularity in response patterns from the collected data. The samples cover 27 provinces such as Shanghai, Tianjin, Zhejiang and Sichuan. A total of 560 questionnaires were distributed. In order to ensure data quality, questionnaires that did not meet the sample requirements were excluded, including questionnaires that were completed within a short time frame, where information was missing, or where the options had significant regularity. After screening, 433 valid questionnaires remained, with a return rate of 77%. The descriptive statistical analysis of the sample is demonstrated in Table 1.

**TABLE 1:** Descriptive statistics of the sample.

Variables	Category	Frequency	Proportion	Variables	Category	Frequency	Proportion
Gender	Male	180	41.57%	Years of work	1–5 years	143	33.03%
	Female	253	58.43%		6–10 years	127	29.33%
Age	Under 25 years	75	17.32%		11–15 years	90	20.79%
	26–30 years	119	27.48%		16–20 years	29	6.70%
	31–35 years	135	31.18%		21–25 years	20	4.62%
	36–40 years	60	13.86%		Over 26 years	24	5.54%
	41–45 years	22	5.08%	Marriage	Married	264	60.97%
	46–50 years	8	1.85%		Unmarried	155	35.80%
	Over 51 years	14	3.23%		Other	14	3.23%
Educational background	High School and below	37	8.55%	Does the spouse work	Yes	235	89.02%
	Tertiary	105	24.25%		No	29	10.98%
	Undergraduate	269	62.12%	–			
	Master and above	22	5.08%				

*N* = 433.

### 3.2. Measures

In this study, the measurement scales used are all derived from established scales in China and abroad and have good content validity. For the scale originally in English, we carried out the procedure rules of two-way translation and back translation in the process of translation into Chinese. All survey items, with the exception of the demographic ones, are evaluated using a 5-point Likert scale, where the values “1” (strongly disagree) to “5” (strongly agree) correspond to the levels of disagreement or agreement.

#### 3.2.1. Family boundary flexibility

The measurement of family boundary flexibility (FBF) matching includes both family flexibility-willingness (FFW) and family flexibility-ability (FFA). The matching value is represented by the absolute value of the two values difference, so that a smaller value represents a higher degree of matching and vice versa. All questions on the FBF scale were created by Matthews et al. (2010), with five questions on family flexibility-ability and four questions on family flexibility-willingness. The Cronbach’s α coefficient for the FFA is 0.80, whereas it is 0.78 for the FFW, both of which meet the reliability requirement.

#### 3.2.2. Family-work enrichment

Family-work enrichment was established by Wayne et al. (2004), which has 4 items. The Cronbach’s α coefficient for the FWE indicates a level of internal consistency reliability at 0.75, thus meeting the reliability requirement.

#### 3.2.3. Family support

Family support was taken by Li and Zhao (2009). There are 10 items in total. The Cronbach’s α coefficient for the Family Support scale reveals a strong level of internal consistency, measuring at 0.88, which meets the established reliability threshold.

#### 3.2.4. Work engagement

Schaufeli et al. (2006) developed the WE scale, which comprises 9 items. It encompasses three dimensions: energy, dedication, and concentration. The Work Engagement scale demonstrates strong internal consistency, with a Cronbach’s α coefficient of 0.90, which meets the reliability criteria.

#### 3.2.5. Control variables

According to the demographic characteristics variables proposed by previous studies that can influence employees’ work engagement, this study mainly selects gender, age, education background, length of service, marital status and spouse’s work status as control variables to limit.

### 3.3. Methodology

Firstly, we addressed the deviation of the ordinary method by considering two aspects, the measurement procedure, and the statistical method. Regarding the measurement procedures, participants were instructed to utilize anonymous methods in order to minimize their guesses about measurement items. In terms of statistical methods, this study used SPSS21.0 to conduct a Harman single-factor test, which examined the common method variance (CMV) of the scale. Secondly, we utilized AMOS24.0 to perform validation factor analysis, thereby evaluating the validity of the scale. Thirdly, we used SPSS21.0 for descriptive statistical analysis, correlation analysis and multi-collinearity tests. Fourthly, we utilized both the hierarchical regression model and Bootstrap methods (with the sample randomly repeated 5,000 times) to examine the main effect, mediating effect, and moderating effect of the study by using SPSS21.0. Finally, we used the PROCESS plug-in of SPSS to examine the mediating effect under moderated conditions.

## 4. Results

### 4.1. Common method variance (CMV)

To assess the potential presence of CMV in the scale, we use the Harman single-factor test by SPSS 21.0. The result demonstrates that the factor loading of the first principal component obtained was 40.53% when setting the characteristic root greater than 1 and without factor rotation, which is less than the 50% threshold recommended by previous studies (Podsakoff et al., 2003). Therefore, based on preliminary analysis, it can be concluded that the present study does not indicate any significant evidence of substantial common method variance, and follow-up research may be conducted.

### 4.2. Validation factor analysis

We conduct CFA using AMOS 24.0 to assess and compare the goodness of fit for each factor model based on the measured data. The analysis results, displayed in Table 2, reveal that when compared to other models, the five-factors model exhibits a highly satisfactory level of data fitting (χ2/df = 1.943, RMSEA = 0.047, CFI = 0.955, GFI = 0.913, NFI = 0.911, NNFI = 0.949). The five factors in this study demonstrate good discriminant validity for further research.

**TABLE 2:** Results of the validation factor analysis.

Model	Factor	χ ^2^	χ ^2^/df	RMSEA	GFI	CFI	NFI	NNFI
Model 1 (five factors)	FFW, FFA, FE, FS, WE	514.804	1.943	0.047	0.913	0.955	0.911	0.949
Model 2 (four factors)	FFW + FFA, FE, FS, WE	525.542	1.954	0.047	0.911	0.953	0.909	0.948
Model 3 (three factors)	FFW + FFA, FE + WE, FS	833.655	3.065	0.069	0.841	0.898	0.856	0.887
Model 4 (two factors)	FFW + FFA + FE + WE, FS	1171.090	4.274	0.087	0.774	0.837	0.798	0.821
Model 5 (one factor)	FFW + FFA + FE + WE + FS	1647.238	5.990	0.107	0.654	0.750	0.716	0.728

*N* = 433; FFW refers to Family Flexibility-Willingness; FFA refers to Family Flexibility-Ability; FE refers to Family-Work Enrichment; FS refers to Family Support; WE refers to Work Engagement; “ + ” indicates factors combination; Models 2–5 are compared with Model 1.

### 4.3. Descriptive statistical analysis and correlation analysis

Table 3 displays the averages, standard deviations, and Pearson correlation coefficients for the variables examined in the study. From the findings presented in Table 3, it is apparent that there are positive correlations between family boundary flexibility matching and both FWE (*r* = −0.134, *p* < 0.01) and WE (*r* = −0.185, *p* < 0.001) (family boundary flexibility matching takes the absolute value of the difference, so a smaller absolute value indicates a greater match, and thus a negative value); it indicates a statistically significant and positive correlation between FS and WE (*r* = 0.571, *p* < 0.001). The results of the correlation analysis are largely consistent with theoretical expectations, and hypotheses 1 and 2 are initially confirmed, providing initial support for the hypothesis testing that followed.

**TABLE 3:** Descriptive statistics and correlation coefficients for each variable.

	1	2	3	4	5	6	7	8	9	10
1. Gender	1									
2. Age	0.009	1								
3. Educational background	0.018	−0.100*	1							
4. Years of work	0.003	0.763***	−0.013	1						
5. Marriage	0.070	−0.295***	−0.062	−0.176***	1					
6. Spouse’s work	0.002	0.285***	0.109*	0.300***	−0.418***	1				
7. FBF	0.068	0.039	−0.054	0.033	0.045	−0.051	1*			
8. FWE	−0.011	0.101*	0.028	0.033	−0.062	−0.077	−0.134**	1		
9. FS	0.026	0.089	0.053	0.059	−0.098*	−0.090	−0.104*	0.743***	1	
10. WE	−0.110*	0.064	0.040	0.083	−0.089	0.034	−0.185***	0.518***	0.571***	1
M	1.58	2.80	2.64	2.39	1.43	0.57	0.41	3.99	3.96	3.62
SD	0.49	1.40	0.71	1.47	0.59	1.60	0.37	0.74	0.75	0.77

*N* = 433; *p < 0.05 **p < 0.01 ***p < 0.001. FBF refers to Family Boundary Flexibility; FWE refers to Family-Work Enrichment; FS refers to Family Support; WE refers to Work Engagement.

### 4.4. Hypothesis testing

#### 4.4.1. Multi-collinearity test

Before we do the regression analysis, we first perform a multi-collinearity test on the variables. We utilized SPSS software to test, focusing on the aspects of variance inflation factor (VIF) and tolerance (TOL). Table 4 presents the outcomes of the test.

**TABLE 4:** Results of multi-collinearity test.

Variables	Tolerance	VIF
Family boundary flexibility	0.982	1.018
Family-work enrichment	0.445	2.246
Family support	0.448	2.230

The dependent variable is work engagement.

The data analysis results reveal that each variable has a TOL value exceeding 0.1, and the VIF values are all less than 5. These findings indicate no multicollinearity problem between the constructs in the data analyzed in this study (Arabameri et al., 2020; Hair et al., 2020; Talwar et al., 2020; Kumar et al., 2021).

#### 4.4.2. Test for main and mediating effects

This paper used SPSS21.0 software to examine the principal effect and intermediate effect in hypothesis 1 and hypothesis 2, respectively by hierarchical regression analysis. Table 5 presents the test’s results. As demonstrated by Model 1 and Model 2, after controlling several control variables, respectively, the finding indicates that employees’ family boundary flexibility can positively effect their WE (β = −0.366, *p* < 0.001), thus supporting H_1_. Model 3 and Model 4 show that matching of FBF also positively effects FWE after adding control variables (β = −0.278, *p* < 0.01). Model 5 displays that FWE significantly improves WE (β = 0.549, *p* < 0.001). Model 6 demonstrates that when including FBF and FWE simultaneously in the equation, the impact of FBF on FE remains significant (β = −0.218, *p* < 0.05), and the impact of FWE on WE is also significant (β = 0.534, *p* < 0.001). As can be seen from models 1–6, family-work enrichment partially mediates the effect of employees’ family boundary flexibilities on their work engagement, so H_2_ is validated (The family boundary flexibility match takes the absolute value of the difference, so a smaller absolute value indicates a greater match, and so the data results in a negative value).

**TABLE 5:** Results of regression analysis of main effects and mediating effects.

Variables	WE						FWE	
	**Model 1**	**Model 2**	**Model 5**	**Model 6**	**Model 7**	**Model 8**	**Model 3**	**Model 4**
**Control variables**								
Gender	−0.162	−0.144	−0.156*	−0.145	−0.182**	−0.185**	−0.012	0.002
Age	−0.007	−0.003	−0.060	−0.056	−0.049	−0.037	0.095*	0.098*
Educational background	0.039	0.031	0.006	0.002	−0.010	−0.008	0.060	0.053
Years of work	0.046	0.049	0.067*	0.068*	0.049	0.038	−0.037	−0.036
Marriage	−0.098	−0.091	−0.044	−0.041	0.006	−0.011	−0.099	−0.093
Spouse’s work	−0.011	−0.015	0.026	0.022	0.044*	0.041	−0.067**	−0.070**
**Independent variable**								
FBF		−0.366***		−0.218*				−0.278**
**Intermediate variables**								
FWE			0.549***	0.534***	0.223***	0.269***		
**Adjustment variables**								
FS					0.436***	0.500***		
**Interaction items**								
FE × FS						−0.088*		
R2	0.026	0.057	0.293	0.304	0.373	0.397	0.033	0.053
ΔR2	0.026	0.031	0.267	0.247	0.373	0.024	0.033	0.020
F	1.624	3.217**	21.987***	20.532***	27.904***	25.876***	2.093*	2.961**

*N* = 433; *p < 0.05 **p < 0.01 ***p < 0.001. FBF refers to Family Boundary Flexibility; FWE refers to Family-Work Enrichment; FS refers to Family Support; WE refers to Work Engagement.

To further test the mediating role played by the family-work enrichment, we also used Model 4 in the PROCESS plug-in and applied the Bootstrap method, showing that the indirect effect of FWE on the association between FBF and WE is −0.1388, with a 95% confidence interval of [−0.2562, −0.0248], excluding 0, thus H_2_ is again validated.

#### 4.4.3. Test for moderating effect

This paper uses hierarchical regression analysis to verify the moderating role of FS on FWE and WE. Table 3 presents the results of analysis. As seen in Model 7 and Model 8, after controlling for the main effects of FWE and FS, the interaction effect between these two variables significantly influences WE (β = −0.088, *p* < 0.05), suggesting that FS plays a moderating role in the link between these two factors, thus H_3_ is validated.

#### 4.4.4. Tests for mediating effect of being moderated

To investigate the mediating role of moderation in our proposed model, we utilize Model 14 of the PROCESS plug-in for SPSS in this study. As indicated in Table 6, when they perceive a higher level of FS, the indirect impact of FWE on employees’ WE is not substantial, with a 95% confidence interval of [−0.0861, 0.2222]. Whereas when employees perceive low levels of family support, their work engagement is significantly impacted by their family-work enrichment. The 95% confidence interval for the impact, excluding 0, is [−0.1388, −0.0075]. The effect values for the two groups are significantly different (*r* = 0.025, *p* < 0.05). The 95% confidence interval for the impact, excluding 0, is [0.0009, 0.0568]. According to the moderated mediation test proposed by Hayes (2015), the test parameter Index = 0.033 and the 95% confidence interval is [0.0043, 0.0716] excluding 0, indicating the mediation effect is moderated. It follows that FS moderates the mediation influence of FWE on the relation between FBF and WE, producing a moderated mediating effect, thus hypothesis 4 is tested.

**TABLE 6:** Results of regression analysis of moderation effects (*N* = 433).

	Moderating effects	Effect	Boot SE	95% CI	
				**Boot LLCI**	**Boot ULCI**
Indirect effects of family support	High family support	−0.025	0.027	−0.0861	0.2222
	Low family support	−0.063	0.034	−0.1388	−0.0075
	Differences	0.025	0.015	0.0009	0.0568

## 5. Discussion

This study focuses on employee resilience and constructs a moderated mediation model from the standpoint of family boundary flexibility, combining personal-environment fit theory, boundary theory, and family system theory. The purpose is to examine the process and boundary conditions of family boundary flexibility on employees’ work engagement. The findings indicate a positive correlation between family boundary flexibility and employees’ work engagement, thus supporting hypothesis 1. This finding aligns with recent studies conducted by researchers, focusing on the role of family motivation in promoting hard work (Menges et al., 2017; Zhang et al., 2020). Furthermore, it contributes to the existing research on work engagement from the family perspective. Additionally, the results demonstrate that family boundary flexibility enhances work engagement through the achievement of home-work enrichment, supporting hypothesis 2. When employees possess a high degree of compatibility between their family flexibility abilities and willingness, it becomes easier for them to transition between their family and work roles, thereby fostering a positive work-family relationship (Greenhaus and Powell, 2006). Furthermore, the findings reveal that family support moderates the relation of family-work enrichment on work engagement, and the mediating role of family-work enrichment, confirming hypotheses 3 and hypotheses 4. Family support serves as a crucial source of assistance and encouragement (Greenhaus and Powell, 2006), enhancing employees’ confidence and ability to deal with work problems, improving their resilience and ability to resist work pressure, thus positively impacting work engagement (Luthans et al., 2010; Kavikondala et al., 2016).

## 6. Conclusion

The objective of this study is to investigate the underlying mechanism through which the flexibility of family boundaries influences work engagement among employees. Using the Person-Environment Fit theory as an overall framework and based on boundary theory, the study explores the effect of the degree of matching employees’ family boundary flexibility willingness and ability on their work engagement in the Chinese context. The findings confirm a positive effect of employees’ family boundary flexibility on their work engagement. Moreover, the study reveals that family-work enrichment partially mediates the link between family boundary flexibility and work engagement. Additionally, Family support not only serves as a moderator in the association between family-work enrichment and work engagement but also moderates the role of family-work enrichment in mediating the impact of family boundary flexibility on work engagement. A high degree of matching family boundary flexibility enables employees to detach themselves from complicated family chores to deal with work tasks and achieve a balance between family and work. This can help employees improve their mental health, better deal with conflict and stress between work and life, juggle family and work responsibilities, enhance employees’ coping ability and adaptability, increase their engagement to work, improve work efficiency and happiness, and thus enhance their resilience. Therefore, harmonious work-family relationship is an important source for employees to enhance resilience. This study offers a fresh perspective and empirical evidence on the relation between family boundary flexibility and work engagement, thereby expanding the current literature on employees’ work engagement and enhancing our comprehension of the intricacies of family-work dynamics. Furthermore, the study provides valuable insights for organizations seeking to enhance employee engagement and productivity. Future research can build upon this study by exploring other sample populations, investigating additional boundary conditions, and utilizing different research methodologies.

### 6.1. Theoretical implications

This paper expands the research of the Person-Environment Fit theory. Previous studies primarily focused on how well the workplace meets individual requirements (Valcour et al., 2011; Tang et al., 2014; Norling and Chopik, 2020), while this study concentrates on the family domain, providing a clearer outlook to foster profound comprehension of the interaction between family and work. The study shows that the matching of employees’ family flexibility-ability and family flexibility-willingness significantly improves employees’ resilience to work challenges, which further increases employees’ work engagement. Such findings echo calls from Voydanoff (2005) and King et al. (2016), while suggesting that future research concentrate more on the positive impact of the family domain on employee resilience.

This paper further explores the influence of individual preference characteristic factors in the research of the family-work interface. Existing research in both domestic and international contexts has primarily concentrated on examining the influence of objective factors within the family and work domains on the work-family interface (Beigi et al., 2018; Piszczek and Berg, 2020). However, there has been less emphasis on the role of individual preference characteristics in this relationship. This study introduces the concept of boundary flexibility and incorporates the element of individuals’ willingness and ability to manage family boundaries and role transitions into the analytical framework, in order to provide a richer and more varied level of research on work-family interaction.

### 6.2. Practical implications

Firstly, companies should give sufficient respect and attention to each employee’s family boundary flexibility and promote its positive impact as much as possible. Only when individuals’ family flexibility ability and willingness reach a higher matching level can they feel the stronger spillover effect of family on work, thus improving work efficiency and performance. To promote work-life balance, organizations should comprehensively understand the degree of employees in managing family flexibility boundary, establish family profiles for employees, and differentiate the management of employees according to their different degrees of family boundary flexibility (Shockley and Allen, 2010). For example, place employees in appropriate positions, allocate time and tasks to employees in a targeted manner, set flexible working hours for employees, and reasonably allocate work-linked behavior during non-working hours.

Secondly, companies can adopt family-friendly policies such as flexible working, setting up childcare centers in the workplace and allowing employees to apply to complete relevant work at home to create a relaxed and comfortable working environment. This approach can foster stronger connections between employees’ family and work domains, leading to benefits such as greater work involvement, reduced turnover, and increased job satisfaction (Menges et al., 2017). These policies not only shorten the distance between employees and the organization and create a good organizational atmosphere, but also effectively alleviate the conflict between employees’ family and work, enabling employees to achieve more satisfaction and happiness, and enhancing their resilience and commitment to work. We also call on companies to create a corporate culture that supports the integration of work and family. When employees feel that their family needs are being cared for and valued by the company, they will take more positive action and work to repay the company’s care for their family needs.

Finally, it is recommended that companies consider the management of personal family boundary flexibility in their employees’ career development plans. When developing talent, it’s important for enterprises to conduct a thorough analysis of job requirements and position characteristics (Boštjančič and Slana, 2018), and to assess the necessary boundary-spanning skills for the role. At the same time, personal family boundary flexibility is included in the scope of investigation to consider whether the personal family boundary flexibility of employees conflicts with the post boundary elasticity. Managers should deeply analyze the policies currently provided by the organization, seek suitable talents, and reduce mismatched efficiency waste for the organization from the source.

### 6.3. Methodological implications

First, this study conducted random sampling of Chinese employees to obtain samples. However, employees with different job types have different boundary management abilities. Future researches suggest that subjects should be classified according to employees’ job types, such as knowledge workers, front-line operational employees.

Second, the cross-sectional data used in this paper may not be sufficient to reveal deep structural relationships. In contrast, the longitudinal multi-point data collection method is more scientifically rigorous. Future studies could consider using longitudinal data to re-model their results by replicating the study.

Finally, we strongly advocate the use of a combination of qualitative and quantitative approaches to research design. In addition, the method of questionnaire survey can be replaced by experimental observation and diary observation, which can help us understand the work-family balance of the subjects more intuitively.

### 6.4. Limitations and future directions

First, increase the diversity of data. We use the self-report scale to collect and measure data in this study. Although the data passed the bias test of common methods, there were inevitably errors caused by subjective responses of the self-rating scale and homologous data. Therefore, future studies can use objective indicators such as family support resources and family members, and the interdependence between employees and their spouses, to improve measurements in the family domain. Furthermore, it may be necessary to diversify the data sources in the future to expand the coverage of samples and increase the applicability of the findings.

Second, enrich the theoretical framework of family boundary elasticity research. This study solely examines the moderating impact of the degree of family support on the relation between family boundary flexibility and employees’ work engagement. Subsequent studies can further investigate the boundary conditions that impact the association between matching family boundary flexibility and employees’ job engagement. Individuals’ family-work values may vary based on cultural and national (Ford et al., 2007; Allen et al., 2014). Unlike the individualistic values that predominate in Western societies (Kossek et al., 2012), Chinese employees in enterprises tend to hold collectivist values (Yan et al., 2023). They take into consideration their family’s needs while pursuing their work objectives. Therefore, future studies should investigate the influence of cultural differences between China and the West on individual family boundary flexibility. In addition, it is also likely to be influenced by organizational factors, including organizational atmosphere and leadership management style.

Third, expand the dynamic research of work-family interaction. This study uses cross-sectional data. However, as Ammons (2013) notes, an individual’s capability and willingness of family boundaries may be subject to change due to factors such as their age and evolving family structure, and the match degree between the individual and their environment may also vary over time. In future studies, the methods of longitudinal studies can be used to track and document family boundary flexibility over a longer range, in order to provide a new perspective for the study of family and work.

## Data availability statement

The raw data supporting the conclusions of this article will be made available by the authors, without undue reservation.

## Author contributions

DY: literatures and writing the manuscript. YL: data analysis and writing the manuscript. HZ: designing the research and reviewing and revising the manuscript. YZ: processing data. All authors contributed to the article and approved the submitted version.

## Footnotes

1
http://www.credamo.com

2
http://www.wjx.cn

3
http://www.wenjuan.com
